# Tri-Band Regulation and Split-Type Smart Photovoltaic Windows for Thermal Modulation of Energy-Saving Buildings in All-Season

**DOI:** 10.1007/s40820-025-01985-w

**Published:** 2026-01-05

**Authors:** Qian Wang, Zongxu Na, Jianfei Gao, Li Yu, Yuanwei Chen, Peng Gao, Yong Ding, Songyuan Dai, Mohammad Khaja Nazeeruddin, Huai Yang

**Affiliations:** 1 Institute for Advanced Materials and Technology, University of Science and Technology Beijing, Beijing, 100083 People’s Republic of China; 2 State Key Laboratory of Green and Efficient Development of Phosphorus Resources, Hubei Key Laboratory of Plasma Chemistry and Advanced Materials, School of Materials Science and Engineering, Wuhan Institute of Technology, No. 206 Guanggu 1 Road, Wuhan, 430205 People’s Republic of China; 3 Fujian Institute of Research on the Structure of Matter, Chinese Academy of Sciences, Fuzhou, 350002 People’s Republic of China; 4 College of Renewable Energy, Hohai University, Changzhou, 213000 People’s Republic of China; 5 Beijing Key Laboratory of Novel Thin-Film Solar Cells, School of New Energy, North China Electric Power University (NCEPU), Beijing, 102206 People’s Republic of China; 6 Institute of Chemical Sciences and Engineering, École Polytechnique Fédérale de Lausanne (EPFL), CH-1015 Lausanne, Switzerland; 7 School of Materials Science and Engineering, Peking University, Beijing, 100871 People’s Republic of China

**Keywords:** Smart photovoltaic windows, Polymer-dispersed liquid crystals, Passive radiative cooling, Tri-band regulation, Energy-saving buildings

## Abstract

Energy-saving buildings (ESBs) are an emerging green technology that can significantly reduce building-associated cooling and heating energy consumption, catering to the desire for carbon neutrality and sustainable development of society. Smart photovoltaic windows (SPWs) offer a promising platform for designing ESBs because they present the capability to regulate and harness solar energy. With frequent outbreaks of extreme weather all over the world, the achievement of exceptional energy-saving effect under different weather conditions is an inevitable trend for the development of ESBs but is hardly achieved via existing SPWs. Here, we substantially reduce the driving voltage of polymer-dispersed liquid crystals (PDLCs) by 28.1 % via molecular engineering while maintaining their high solar transmittance (*T*_sol_ = 83.8 %, transparent state) and solar modulating ability (Δ*T*_sol_ = 80.5 %). By the assembly of perovskite solar cell and a broadband thermal-managing unit encompassing the electrical-responsive PDLCs, transparent high-emissivity SiO_2_ passive radiation-cooling, and Ag low-emissivity layers possesses, we present a tri-band regulation and split-type SPW possessing superb energy-saving effect in all-season. The perovskite solar cell can produce the electric power to stimulate the electrical-responsive behavior of the PDLCs, endowing the SPWs zero-energy input solar energy regulating characteristic, and compensate the daily energy consumption needed for ESBs. Moreover, the scalable manufacturing technology holds a great potential for the real-world applications.

**Supplementary Information:**

The online version contains supplementary material available at 10.1007/s40820-025-01985-w.

## Highlights

- Broadening the modulation range and decreasing the driving voltage of polymer dispersed liquid crystals via molecular engineering without sacrificing high solar transmittance (transparent state) and solar modulating ability.
- Modulating capability of the smart photovoltaic windows across visible, near-infrared and mid-infrared bands enabling superb energy-saving performance in all season.
- Holding a great potential for real-world application due to their scalable manufacturing technology.

**Supplementary Information:**

The online version contains supplementary material available at 10.1007/s40820-025-01985-w.

## Graphical abstract

**Figure:**
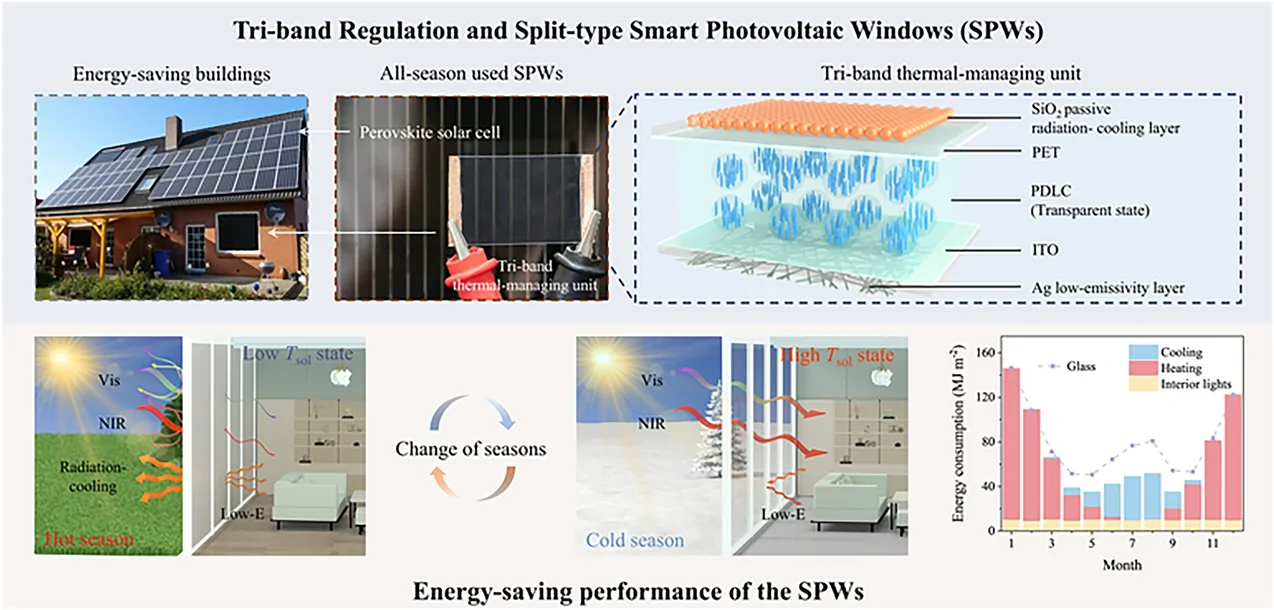

## Introduction

Building energy consumption approximately occupies 30 % of global energy [1, 2]. With frequent outbreaks of extreme weather all over the world, the building-associated cooling and heating energy consumption increases year by year [3]. Energy-saving buildings (ESBs), a green technology capable of effectively restraining the energy consumption via regulating and harnessing solar energy, emerge as the times require [4].

Windows are the primary medium for the energy exchange between ESBs and external environment [5]. The proportion of windows in modern architecture is gradually increasing [6]. Making windows active to react to the weather condition of external environment affords an effectual avenue for exploiting ESBs [7]. Smart photovoltaic windows (SPWs), as a multifunctional stimuli-responsive device, offer a promising platform for exploring ESBs attributed to their significant features [8, 9]. Their transparency can be dynamically modulated on demand under a variety of external stimuli to enable ESBs the energy-saving effect [10–13]. They are able to directly convert solar energy into electrical energy providing additional energy source for daily consumption of ESBs [14].

To date, the combination of electrical-responsive chromic materials and the photovoltaics has become the mainstream for designing SPWs for several reasons. The electrical-responsive feature enables SPWs to freely modulate solar energy according to the demand of the occupants under complex environments [15–17]. The electric output generated by the photovoltaics via harnessing solar energy is able to be utilized for trigger dynamic solar energy regulating behavior of electrical-responsive chromic materials which endows the SPWs self-driven characteristics, further enhancing their energy-saving performance [18]. In addition, electrical-responsive feature makes SPWs facile to be connected with the computer and internet equipment holding the potential for constructing smart home systems in the future [19].

Diverse electrical-responsive chromic materials including conducting polymers [20, 21], viologens [22, 23], transition metal oxides [24–30], and liquid crystals [31, 32] have been employed as a chromic unit to assemble with the photovoltaics such as organic, dye-sensitized, silicon, and perovskite solar cells for developing SPWs of distinct micro-/macrostructures. Compared to other electrical-responsive chromic materials, polymer-dispersed liquid crystals (PDLCs) have been considered as an ideal candidate for exploiting SPWs because they can be mass manufactured into large-scale flexible films that possess high contrast ratio, fast response speed, and excellent cyclical stability [33]. Reducing the driving voltage and improving the solar modulating ability (Δ*T*_sol_) are key issues for real-word application of PDLCs in SPWs [34]. Recently, the doping of nanoparticles, BaTiO_3_, ZnO, and Ag, into PDLCs is a commonly used and effective strategy for lowering the driving voltage [35–37]. However, the transmittance of PDLCs at transparent state inevitably sacrifices in order to significantly reduce the driving voltage due to scattering effect of the nanoparticles, which is adverse to the realization of extraordinary energy-saving effect in all-season and their wide applications across distinct climate zones [38, 39]. According to the energy distribution of solar irradiation and the spontaneous emission, a broadband regulating capability in the region of visible, near-infrared, and mid-infrared is required for achieving ideal energy-saving performance [40]. For this, previous researches have introduced fluorine-containing monomer into PDLCs to enable dynamic thermal modulating performance in visible and near-infrared area and enhance their passive radiation-cooling effect [41–43]. However, the application of fluorine-containing compounds certainly will cause serious harm to environment and human health [44].

**Fig. 1:**
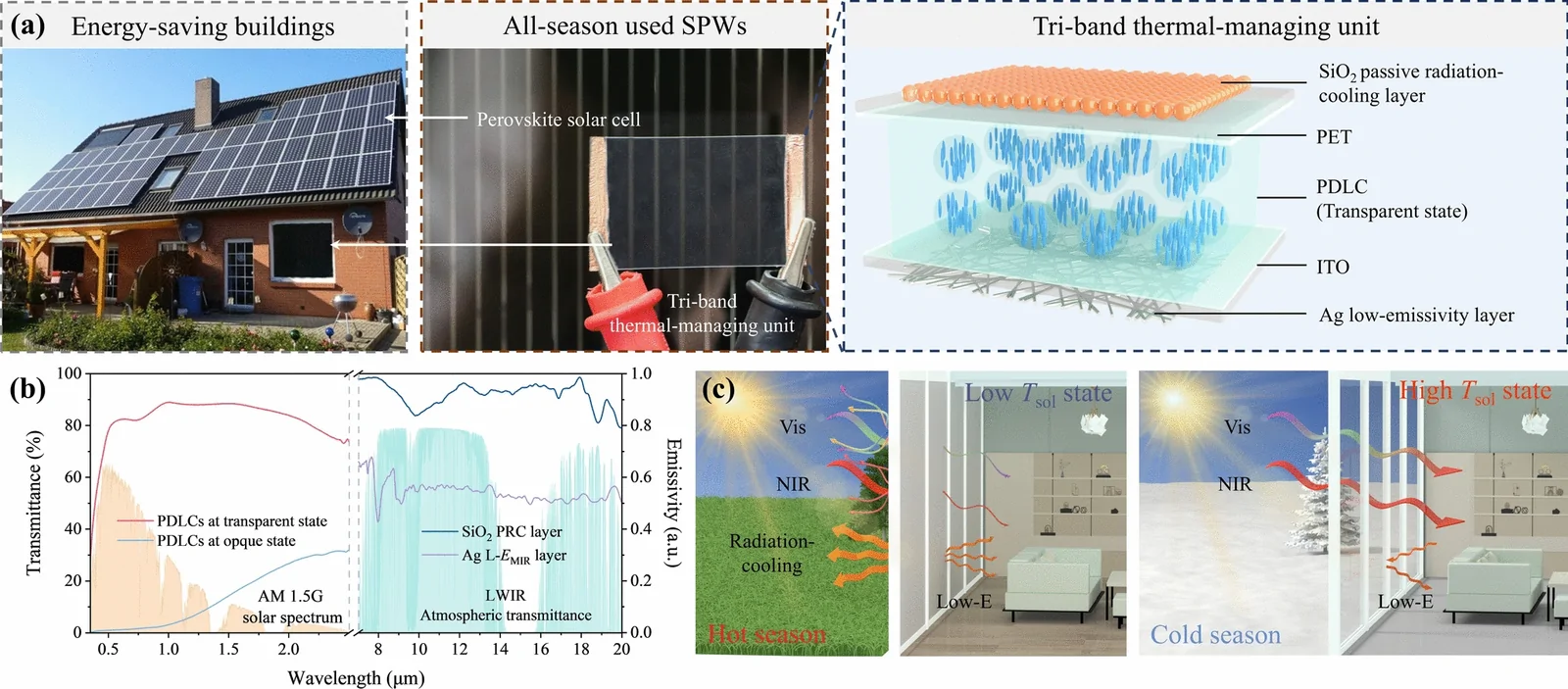
**a** Photographs of the tri-band regulation and split-type SPWs for all-season used ESBs, along with a diagrammatic sketch illustrating the architecture of the BTMU. **b** Transmittance spectra of (0.38–2.5 µm) of the PDLCs at transparent and opaque states, and MIR emissivity (7.0–20 µm) of the SiO_2_ PRC and Ag layers in optimized BTMU. **c** Working principle of the SPWs in hot season (left) and cold season (right)

Herein, we remarkably reduce the driving voltage via introducing polar and nonpolar molecules into the PDLCs while maintaining their high solar transmittance (transparent state) and Δ*T*_sol_. A broadband thermal-managing unit (BTMU) is built up by electrical-responsive PDLCs, transparent high-emissivity (H-*E*_MIR_) SiO_2_ passive radiation-cooling (PRC), and Ag low-emissivity (L-*E*_MIR_) layers (Fig. 1a). By assembling the BTMU and perovskite solar cell (Table S1), we present a conceptional demonstration of a tri-band regulation and split-type SPW for all-season ESB thermal regulation. Unlike previously reported SPWs [20–32], our designed SPWs possess broadband modulating ability to visible (380–780 nm), near-infrared (NIR, 780–2500 nm), and mid-infrared (MIR, 8–13 µm) enabling exceptional energy-saving effect in all-season (Fig. 1b). As presented in Fig. 1c, in hot season, the PDLCs at opaque state can efficaciously block the thermal radiation of visible and NIR bands in solar energy into the indoor while SiO_2_ layer displays passive radiative cooling effect, which is able to greatly reduce the cooling energy consumption [45, 46]. In cold season, the thermal radiation of visible and NIR bands in solar energy is allowed for entering into the indoor to compensate the energy consumption needed for the heating thanks to high solar transmittance (*T*_sol_) of the BTMU when the PDLC is at transparent state (Fig. S1). In addition, Ag L-*E*_MIR_ layer is beneficial for suppressing the radiative heat exchange between the indoor and outdoor environments in all-season further elevating the energy-saving effect of the SPWs [47]. Besides superb energy-saving performance in different seasons, two significant features make our designed SPWs broad prospects in ESBs. The perovskite solar cell is capable of providing the SPWs high photoelectric conversion efficiency (PCE) and powering the stimuli-responsive behavior of the PDLCs by harnessing solar energy. Therefore, tri-band regulation characteristic of the SPWs for achieving energy-saving effect in all-season requires none-energy input. Moreover, the SPWs are potential for industrial mass production because they are manufactured mainly using roll-to-roll and spraying technologies.

## Experimental Section

### Materials

The polymer monomer and small molecule liquid crystal (SMLC) used for synthesizing the PDLCs were purchased from Yantai Xian Hua Technology Group Co., Ltd. Hydrophobic fumed silica, AEROSIL R812S, was obtained from Evonik. Isopropanol, butyl acetate, ethanol, and tetrahydrofuran (THF) were purchased from Sinopharm Chemical Reagent Co., Ltd. Poly(ethylene-co-acrylic acid) with 15 wt% acrylic acid content (EAA) were purchased from Aladdin. All chemicals were used as received.

### Preparation of Polymer-Dispersed Liquid Crystals (PDLCs)

The polymer monomer, SMLC, photo initiator, and glass micro balloon were well mixed and served as the syrup. The syrup was injected between two conductive PET films. The PDLCs were obtained via roll-to-roll process and exposure to ultraviolet light (365 nm, 15 mW cm^−2^) for 15 min. The thickness of the PDLCs was 20 μm and controlled by the diameter of the glass micro balloon. The composition of the PDLCs is seen in Table S3. The PDLCs with different concentrations of the POSS were prepared by the similar process in which the POSS was mixed with the polymer monomer, SMLC, photo initiator, and glass micro balloon and served as the syrup (Table S4).

### Preparation of Broadband Thermal-Managing Unit (BTMU)

To make sure good adhesion of SiO_2_ passive radiation-cooling and Ag low-emissivity layers to the PDLCs, the EAA, as an adhesive layer, was coated on upper and lower surfaces of the PDLC. Hydrophobic fumed SiO_2_ was dispersed in 100 mL isopropanol. To fabricate the BTMU, hydrophobic fumed silica nanoparticles and laboratory-synthesized Ag nanowires were firstly dispersed into the solvent of isopropanol and ethanol, respectively. The concentration of the silica nanoparticle and Ag nanowire dispersion solution was 10 and 3 mg mL^−1^. The BTMU was prepared by spraying the silica nanoparticle and Ag nanowire dispersion solution on upper and lower surfaces of the PDLCs. The spraying process was conducted using a spray gun with the pressure of 0.2 MPa and moving speed of 7 cm s^−1^, following a line-to line fashion.

### Characterizations

The Vis–NIR transmittance spectra for all samples were measured by using a UV–Vis–NIR spectrophotometer (PerkinElmer, Lambda 1050 +). The solar transmittance (*T*_sol_) is calculated as follows:

1 $$T_{{{\mathrm{sol}}}} = \frac{{\mathop \smallint \nolimits_{0.3 \mu m}^{2.5 \mu m} T\left( \lambda \right)\psi_{{{\mathrm{sol}}}} \left( \lambda \right){\mathrm{d}}\lambda }}{{\mathop \smallint \nolimits_{0.3 \mu m}^{2.5 \mu m} \psi_{{{\mathrm{sol}}}} \left( \lambda \right){\mathrm{d}}\lambda }}$$

where *T*(*λ*) is the measured spectral transmittance and ψ_sol_(*λ*) is the spectral solar power (AM1.5G).

The electro-optical properties of the PDLCs were characterized using a LC parameter analyzer (LCT-5016C, Changchun Liancheng Instrument Co., Ltd.). A halogen-based laser source emitting at 550 nm and a 100 Hz square-wave electric field were applied during the measurement. The field emission scanning electron microscope (FE-SEM, Carl Zeiss, SUPRA 55 SAPPHIRE) was used to characterize the microstructure of the PDLCs, high-emissivity (H-*E*_MIR_) SiO_2_ passive radiation-cooling (PRC) and Ag low-emissivity (L-*E*_MIR_) layers, and morphology of laboratory-synthesized Ag nanowires.

Emissivity curves across the 2.5–25 μm spectral band were measured via a FTIR spectrometer (INVENIO-S, Bruker) integrated with a gold-plated integrating sphere. Following Kirchhoff’s radiation principle, which states *α*(*λ*) = *ε*(*λ*) at thermal equilibrium, the emissivity was calculated using *ε*(*λ*) = 1 − *ρ*(*λ*) − *τ*(*λ*). The infrared camera (FLIR E54) was used to characterize the passive radiation-cooling effect of the SiO_2_ passive radiation-cooling PRC layer and heat insulation performance of the Ag L-*E*_MIR_ layer.

### Energy-Saving Performance Simulation

In EnergyPlus, a model house measuring 8 m (*L*) × 8 m (*W*) × 3 m (*H*) was established, with 4 m × 2 m windows centrally located on each wall, resulting in a window-to-wall ratio of 33 % (Fig. S15). The heating and cooling energy consumption was modeled using EnergyPlus whole-building energy simulation by applying an “ideal-loads-air-system” supplied by district cooling and heating sources. For cooling, the indoor temperature was maintained at 24 °C, and for heating, it was maintained at 22 °C. An air change rate 0.3 ACH (air changes per hour), which is consistent with the requirement of both ASHRAE 90.1 and ASHRAE 62.1 standards, was selected as the air infiltration parameter. The annual electricity generation of perovskite solar cell was calculated using EnergyPlus built-in photovoltaic function, with the area set to 50 % of the roof area, a conversion efficiency of 16 %, and a transmission efficiency of 98 %. In the carbon emission calculations, the carbon emission factor for electricity was set to 0.84 kgCO_2_ kWh^−1^, and for natural gas, it was set to 0.056 kgCO_2_ MJ^−1^.

## Results and Discussion

### Modulation the Electro-Optical Properties of PDLCs via Molecular Engineering

**Fig. 2:**
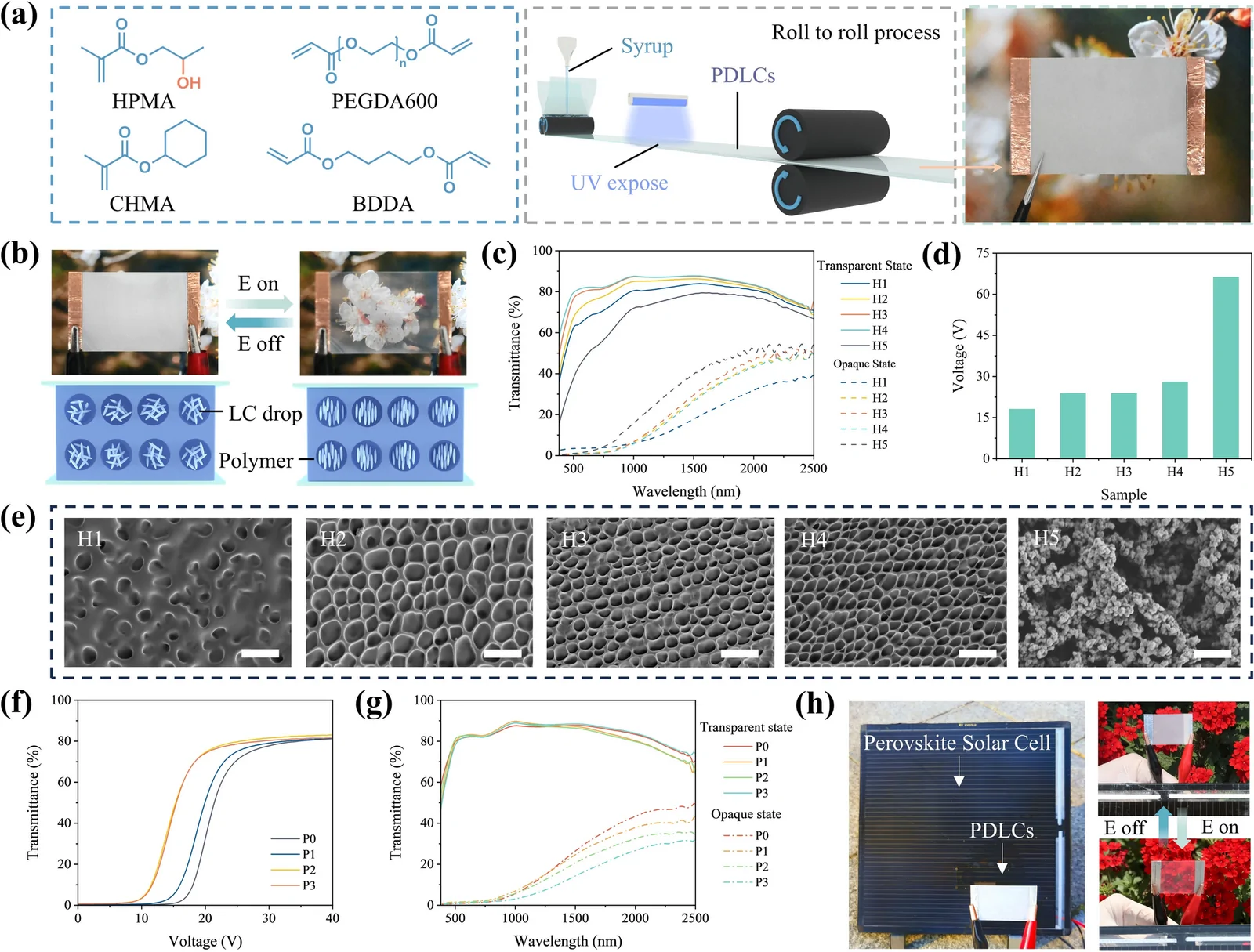
**a** Chemical structure of the polymer monomer using for fabricating low-voltage driven, fast response, fatigue resistant, and visible and NIR modulable PDLCs (left). Schematic illustration of the roll-to-roll technology for fabricating the PDLCs (middle). Photograph of as-prepared PDLCs (right). **b** Photographs of the PDLCs under the voltage of 0 V and 30 V (top). Schematic illustration of the alignment of the SMLC in the PDLCs at transparent and opaque states (bottom). **c** Transmittance spectra of (0.38–2.5 µm) of the PDLCs with different contents of the A-HG at transparent (0 V) and opaque states (30 V). **d** Saturation voltage (V_*sat*_, driving voltage) and **e** scanning electron microscope (SEM) images of the PDLCs with different contents of the A-HG. The scale bar is 6 µm. **f** Electro-optical curve of the PDLCs with different contents of the POSS. **g** Transmittance spectra of (0.38–2.5 µm) of the PDLCs with different contents of the POSS at transparent (0 V) and opaque states (30 V). **h** Self-powered feature of the PDLCs

To prove our concept, we initially fabricate low-voltage driven, fast response, fatigue resistant, and visible and NIR modulable PDLCs by utilizing acrylate containing hydroxyl group (A-HG) and E8 as the polymer monomer and small molecule liquid crystal (SMLC) through roll-to-roll technology (Fig. 2a). Under external electric field, the PDLCs can reversibly switch between transparent and opaque states due to the alteration of the alignment of the SMLC (Fig. 2b and Movie S1) [48, 49]. As presented in Figs. 2c, S2a and Movie S2, the PDLCs exhibit low *T*_sol_ at initial state (opaque state) because randomly aligned SMLC leads the mismatch in refractive indexes between the polymer matrix and SMLC [50]. On the contrary, the PDLCs display high *T*_sol_ as applying voltage (transparent state) because the external electric field can induce vertical alignment of the SMLC making the ordinary refractive index of the SMLC match with the refractive index of the polymer matrix [51]. Obviously, the content of the A-HG has a significant impact on the *T*_sol_ of the PDLCs at both transparent and opaque states that determines their solar modulating ability (Δ*T*_sol_) (Fig. S2b, c) [52]. When the content of the A-HG is lower than 10.05 wt%, the transmittance of the PDLCs in visible region apparently improves while that of the PDLCs in NIR region clearly drops with the increasing content of the A-HG owing to the reduction of the size of SMLC droplets (Figs. 2e-(ⅰ-ⅳ) and S3) [53]. The smaller size of the SMLC droplets is, the higher saturation voltage (*V*_*sat*_, driving voltage) is required for inducing vertical alignment of the SMLC in the PDLCs because of higher anchoring energy exerted on the SMLC resulting from increased interaction area between the SMLC droplets and polymer matrix (Fig. 2d) [54]. When the content of the A-HG further increases to 13.43 wt%, the SMLC, as a continuous phase instead of the droplets, disperses in the PDLCs which greatly sacrifices the Δ*T*_sol_ and raises the driving voltage of the PDLCs (Fig. 2d, e-ⅴ). Although the PDLCs possess outstanding Δ*T*_sol_ of 79.49 % as the content of the A-HG is 10.05 wt%, the driving voltage of 28.1 V is relatively higher which is inconvenient to integrate with perovskite solar cell for realizing the self-powered feature. In order to diminish the driving voltage of the PDLCs, and meanwhile maintain excellent solar modulating ability, a cage-molecule polyhedral oligomeric silsesquioxane (POSS) is introduced into the PDLCs (Fig. S4). As presented in Figs. 2f and S5a, the driving voltage of the PDLCs substantially declines from 28.1 to 20.2 V as the concentration of the POSS is 3 wt% because the incorporation of the nonpolar POSS with low surface energy can decrease the anchoring energy of the polymer matrix to the SMLC and enhance the steric repulsion between the SMLC and polymer matrix (Fig. S5b) [55, 56]. Simultaneously, the Δ*T*_sol_ of the PDLCs is as high as 80.5 % when the concentration of the POSS is 3 wt% indicating that the addition of the POSS does not sacrifice the solar modulating ability because original microstructure of the PDLCs and size of the SMLC droplets are well retained (Figs. 2g and S5c, d). The high Δ*T*_sol_ is promising for providing SPWs superb energy-saving performance (Table S2). Thanks to the low driving voltage of the PDLCs, perovskite solar cell is capable of affording a steady output voltage to stimulate the electrical-responsive behavior of the PDLCs during the day time for providing the energy-saving effect (Figs. 2h, S6, and Movie S3). Last but not the least, the PDLCs exhibit fast response characteristic, excellent cyclical, and environmental stability that are important for their practical application (Figs. S7, S8 and Movie S4). Notably, 95.2 % initial power conversion efficiency of the perovskite solar cell still retains after it powers the transparency switching behavior of the PDLCs for 1500 cycles (Fig. S9).

### Construction and Optimization of Mid-Infrared Radiation Modulation Layers

**Fig. 3:**
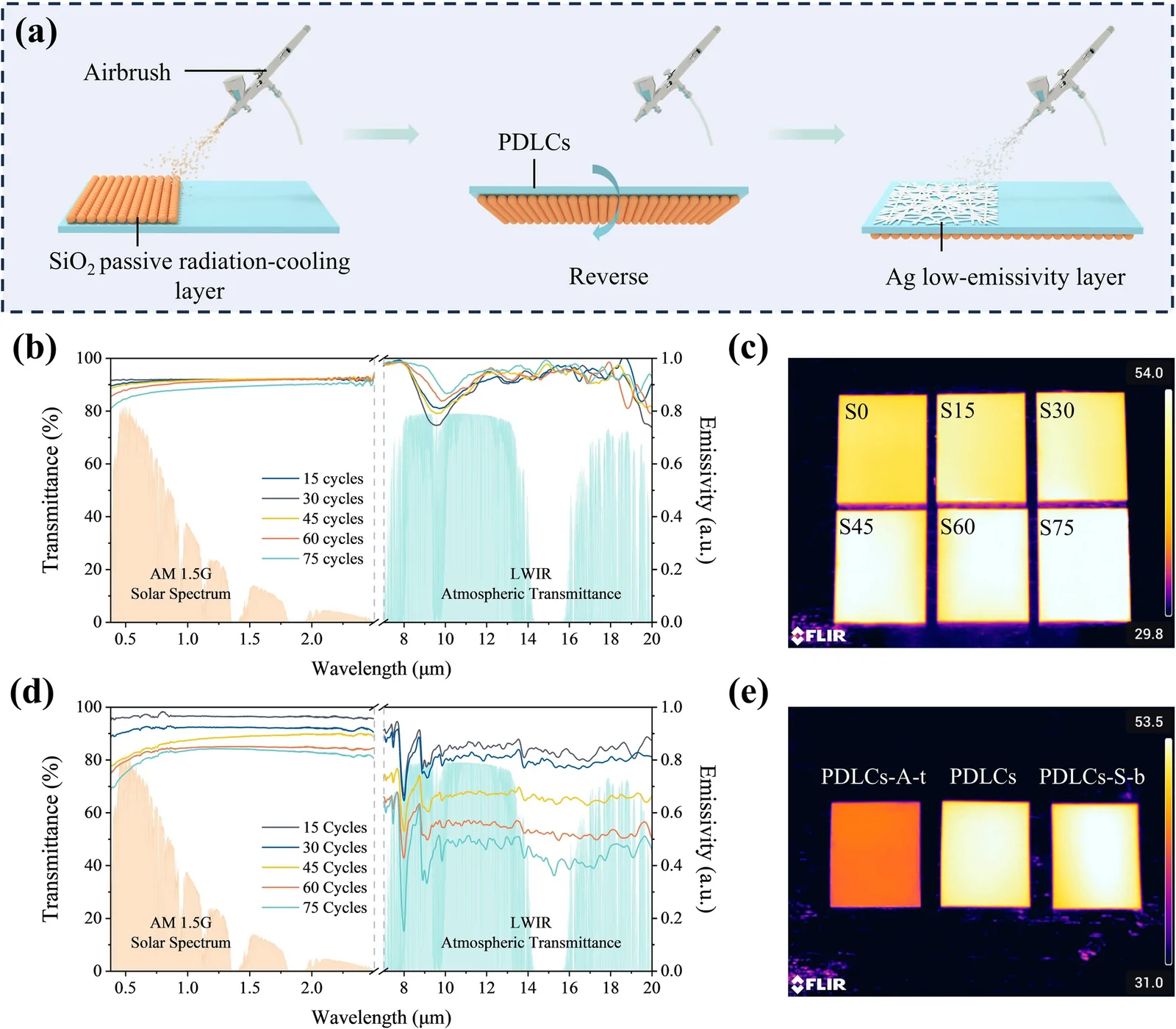
**a** Schematic illustration of the spraying technology for fabricating the BTMU. **b** Transmittance spectra of (0.38–2.5 µm) and MIR emissivity (7.0–20 µm) of the SiO_2_ PRC layer prepared by different spraying cycle numbers. **c** IR images of the SiO_2_ PRC layer of different spraying cycle numbers on a hot stage with the temperature of 60 °C. **d** Transmittance spectra of (0.38–2.5 µm) and MIR emissivity (7.0–20 µm) of Ag L-*E*_MIR_ layer prepared by different spraying cycle numbers. **e** IR images of PDLCs with top surface coated by Ag L-*E*_MIR_ layer (60 spraying cycles, PDLCs-A-t), PDLCs, and PDLCs with bottom surface coated by SiO_2_ PRC layer (60 spraying cycles, PDLCs-S-b) on a hot stage with the temperature of 60 °C

To endow with the SPWs ideal energy-saving effect in all-season which is hardly achieved in the existing SPWs [20–32], a BTMU encompassing the electrical-responsive PDLC, H-*E*_MIR_ SiO_2_ PRC layer, and Ag L-*E*_MIR_ layer is designed according to broadband thermal radiation of solar energy and fabricated by coating the SiO_2_ nanoparticles and Ag nanowires on upper and lower surfaces of the PDLCs via spraying technology (Fig. 3a). Herein, SiO_2_ nanoparticles are selected to build up the PRC layer because of their low absorbance in visible and NIR region, H-*E*_MIR_ in MIR region, and modulable microstructure [57, 58]. The laboratory-synthesized Ag nanowires are employed to fabricate the L-*E*_MIR_ layer originates because it is facile to form the microstructure of high transparence in visible and NIR region and high reflectance in MIR region based on them (Fig. S10) [59, 60]. In our design, the working principle of the BTMU offering the SPWs energy-saving effect is based on the regulating capability of its three key components to solar energy. In hot season, the PDLCs at opaque state effectively block visible and NIR light entering into the indoor and H-*E*_MIR_ SiO_2_ PRC layer continuously radiates the heat through atmospheric window (8–13 µm), which can significantly reduce the energy consumption needed for the air-conditioning. In cold season, high *T*_sol_ of the BTMU (the PDLCs is at transparent state) is conducive to the thermal radiation of solar energy into the indoor, and thus saving the energy consumption demanded for the heating. In both hot and cold seasons, the Ag L-*E*_MIR_ layer can curb the radiative heat exchange between the indoor and outdoor environments further enhancing the energy-saving effect of the SPWs. Consequently, SiO_2_ PRC layer of high H-*E*_MIR_ and *T*_sol_ and Ag layer of L-*E*_MIR_ and high *T*_sol_ are required for enabling SPWs superb energy-saving effect in all-season. As presented in Figs. 3b, c, and S11a, the increment of the number of spraying cycles can raise the *E*_MIR_ of the SiO_2_ PRC layer caused by the augment of the absorption coefficient, and resulting in the rise of the radiative cooling effect [61]. However, larger number of spraying cycles inevitably lead the formation of SiO_2_ agglomerates in micrometer size which lowers the *T*_sol_ of the SiO_2_ PRC layer because of the scattering effect (Figs. 3b and S11b, c). Similarly, growing number of spraying cycles can diminish the *E*_MIR_ of Ag layer enhancing the heat-retaining capacity but sacrifice the *T*_sol_ owing to the denser structure (Figs. 3d, e, and S12) [62]. To balance the trade-off between the *E*_MIR_ and *T*_sol_ of the SiO_2_ PRC and Ag layers for giving the SPWs optimized energy-saving effect in both hot and cold seasons, the SiO_2_ PRC and Ag layers made from 60 spraying cycles are finally utilized to integrate with the PDLCs for the construction of the BTMU. To elucidate operational stability of the SPWs for real-world application, a series of accelerated aging tests are performed on the SiO_2_ PRC and Ag layers. The *E*_MIR_ of the SiO_2_ PRC and Ag layers almost retain for the accelerated aging tests, proving their superior environmental stability (Fig. S13).

### Energy‑Saving Potential Simulation of SPWs

**Fig. 4:**
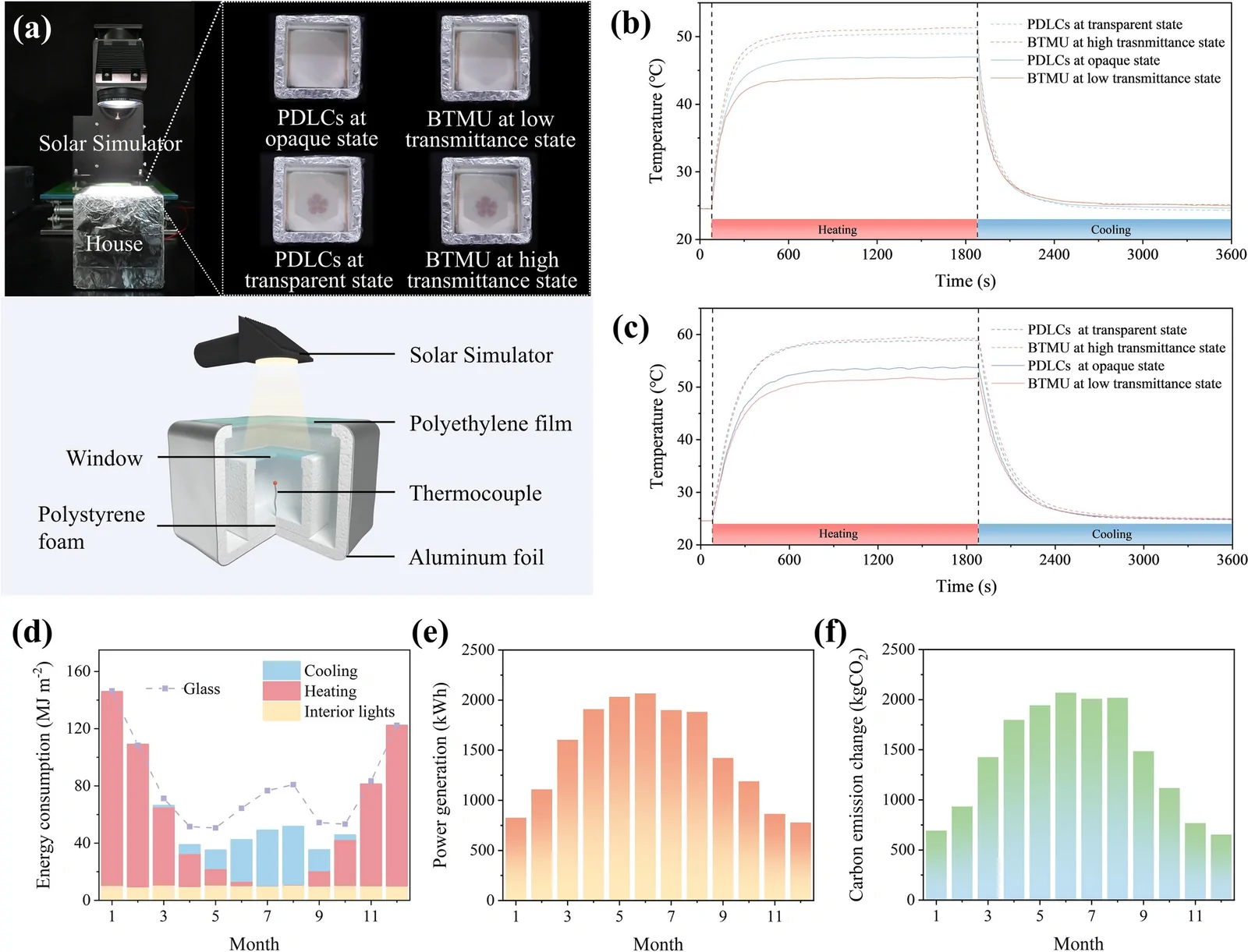
**a** Photograph and diagrammatic sketch of the apparatus for evaluating the energy-saving performance of the SPWs. Chamber temperature variation curves during the process of solar irradiation and unirradiation under different states of the window (BTMU and PDLCs) when utilizing using **b** xenon and **c** infrared lamps as the solar simulator (HT: high *T*_sol_ state; LT: low *T*_sol_ state; IX: illumination by the xenon lamp; N: non illumination). **d** Monthly energy consumption of the SPWs and normal glass window in the climate condition of Beijing. **e** Monthly power generation of the SPWs according the working mode of the SPWs. **f** Monthly saving carbon dioxide emission estimated based on saving energy consumption and power generation of the SPWs

For evaluating the energy-saving performance of the SPWs, a small “house” is built up by utilizing an aluminum foil wrapped polystyrene foam box and BTMU as the main body and window, respectively (Fig. 4a). The chamber in the polystyrene foam box can be considered as the indoor environment. Its temperature is recorded by a K-type thermocouple. In order to minimize the thermal conduction and convection between the indoor and external environments, the polystyrene foam box is sealed by a polyethylene film. In addition, xenon and infrared lamps are applied for emulating sunlight. As presented in Fig. 4b, the chamber temperature sharply goes up and reaches as high as *T*_BTMU, HT-IX_ = 51.3 °C under the illumination of the xenon lamp when the BTMU exhibits high *T*_sol_. When removing the xenon lamp, the chamber temperature gradually decreases to *T*_BTMU, HT-N_ = 25.1 °C. The large difference of the chamber temperature between the illuminating and nonilluminating states demonstrates exceptional energy-saving effect of the SPWs in cold season. Under the same irradiation condition, the chamber temperature can greatly reduce from *T*_BTMU, HT-IX_ = 51.3 °C to *T*_BTMU, LT-IX_ = 47.0 °C as the BTMU presents low *T*_sol_ verifying superb energy-saving effect of the SPWs in hot season. To validate the PRC effect of H-*E*_MIR_ SiO_2_ layer and heat insulation function of the Ag L-*E*_MIR_ layer, the PDLCs are also employed as the window as the reference. Attributed to the heat insulation function of the Ag L-*E*_MIR_ layer, up on exposure to xenon lamp, the chamber temperature rises from the room temperature to *T*_PDLCs, HT-IX_ = 49.8 °C that is lower than *T*_BTMU, HT-IX_ = 51.3 °C although the *T*_sol_ of PDLCs is higher than that of the BTMU [63]. Under the illumination of the xenon lamp, the chamber temperature is *T*_PDLCs, LT-IX_ = 47.0 °C, which is 3.2 °C higher than *T*_BTMU, LT-IX_ = 43.8 °C when the PDLCs are at opaque state. As a result, the chamber temperature is able to further reduce as irradiated by the xenon lamp with the help of the PRC effect of H-*E*_MIR_ SiO_2_ layer. Obviously, the H-*E*_MIR_ SiO_2_ and Ag L-*E*_MIR_ layers take an essential role in giving the SPWs distinguished energy-saving performance in all-season which is furthered proved in the investigation of the energy-saving effect of the SPWs using an infrared lamp as the solar simulator (Fig. 4c). As Beijing is a large city with great energy consumption, it is chosen as the location of the simulation for energy-saving evaluation (more details about the simulation are given in Experimental Section). With the normal glass as the baseline, the designed SPWs can save 16.7 % of the annual building energy consumption for the heating and air-conditioning (Fig. 4d). In our designed SPWs, the perovskite solar cell is capable of generating the electric energy to stimulate the high *T*_sol_ state of the BTMU, promising the thermal irradiation of solar energy into the indoor and significantly diminishing the heating energy consumption needed in cold season. In hot season, original low *T*_sol_ state of the BTMU is able to effectively hinder the thermal irradiation of solar energy, greatly reducing the energy consumption required for air-conditioning. Therefore, the electric energy produced by the SPWs can be used for daily energy consumption of the ESBs. According to the working mode of the SPWs, the annual average power generation of the SPWs is estimated to be 1.76 × 10^4^ kWh (Fig. 4e). Benefitting from the energy-saving and electric power generation characteristics, the application of the SPWs in the ESBs can approximately reduce 16.9 tons of carbon emissions every year (Fig. 4f). To investigate the effect of the climate condition on the energy-saving performance of our designed SPWs, Oslo, a city of typical temperate marine climate located at high-latitude region, is also selected as the location for the simulation. As presented in Fig. S14a, the designed SPWs can save 7.3 % of the annual building energy consumption. The attenuation of the energy-saving performance for Oslo mainly originates from two reasons. The temperature of Oslo through the year is lower than that of Beijing, especially in hot season. In addition, the solar radiation of Oslo is less than that of Beijing due to the geographic location and climate condition of Oslo, which is proved by annual average power generation of the two cities (Oslo, 1.15 × 10^4^ kWh and Beijing, 1.76 × 10^4^ kWh) (Figs. 4e and S14b). The SPWs can reduce around 10.7 tons of carbon emissions every year as they are employed in Oslo (Fig. S14c).

## Conclusions

In summary, we have demonstrated a tri-band regulation and split-type SPW by assembling a BTMU and perovskite solar cell. The BTMU composed of the electrical-responsive PDLC, H-*E*_MIR_ SiO_2_ PRC and Ag L-*E*_MIR_ layers presents broadband modulating capability in visible, NIR, and MIR region, enabling the SPWs distinguished energy-saving effect in both hot and cold seasons. Our concept could create a broad prospect for real-world application of ESBs, and push forward the realization of the target of carbon neutrality and social sustainable development.

## Supplementary Information

Below is the link to the electronic supplementary material.

Supplementary file1 (MP4 700 KB)

Supplementary file2 (MP4 1616 KB)

Supplementary file3 (MP4 5200 KB)

Supplementary file4 (MP4 1951 KB)

Supplementary file5 (DOCX 6083 KB)
