# Supplementary material for: Tri-Band Regulation and Split-Type Smart Photovoltaic Windows for Thermal Modulation of Energy-Saving Buildings in All-Season
Source: Nanomicro Lett. 2026 Jan 5;18:132. doi: 10.1007/s40820-025-01985-w (PMC12765763; doi:10.1007/s40820-025-01985-w)
Supplement: Supplementary file 5 — Supplementary file5 (DOCX 6083 KB) [file 40820_2025_1985_MOESM5_ESM.docx]

Supporting Information for

**Tri-Band Regulation and Split-Type Smart Photovoltaic Windows for Thermal Modulation of Energy-Saving Buildings in All-Season**

Qian Wang^1^, Zongxu Na^1^, Jianfei Gao^1^, Li Yu^2,^ *, Yuanwei Chen^1^, Peng Gao^3^, Yong Ding^4,^ *, Songyuan Dai^5^, Mohammad Khaja Nazeeruddin^6,^ * and Huai Yang^7,^ *

^1^ Institute for Advanced Materials and Technology, University of Science and Technology Beijing, Beijing 100083, P. R. China

^2^ Hubei Key Laboratory of Plasma Chemistry and Advanced Materials, School of Materials Science and Engineering, Key Laboratory of Green Chemical Engineering Process of Ministry of Education, Wuhan Institute of Technology, No. 206 Guanggu 1st Road, Wuhan 430205, P. R. China

^3^ Fujian Institute of Research on the Structure of Matter, Chinese Academy of Sciences, Fuzhou 350002, P. R. China

^4^ College of Renewable Energy, Hohai University, Changzhou 213000, P. R. China

^5^ Beijing Key Laboratory of Novel Thin-Film Solar Cells, School of New Energy, North China Electric Power University (NCEPU), Beijing 102206, P. R. China

^6^ Institute of Chemical Sciences and Engineering, École Polytechnique Fédérale de Lausanne (EPFL), Lausanne CH-1015, Switzerland

^7^ School of Materials Science and Engineering, Peking University, Beijing 100871, P. R. China

*Corresponding authors. E-mail: [li.yu@wit.edu.cn](mailto:li.yu@wit.edu.cn) (Li Yu); [yding@hhu.edu.cn](mailto:yding@hhu.edu.cn) (Yong Ding); [mdkhaja.nazeeruddin@epfl.ch](mailto:mdkhaja.nazeeruddin@epfl.ch) (Mohammad Khaja Nazeeruddin); [yanghuai@pku.edu.cn](mailto:yanghuai@pku.edu.cn) (Huai Yang)

**Supplementary Figures**


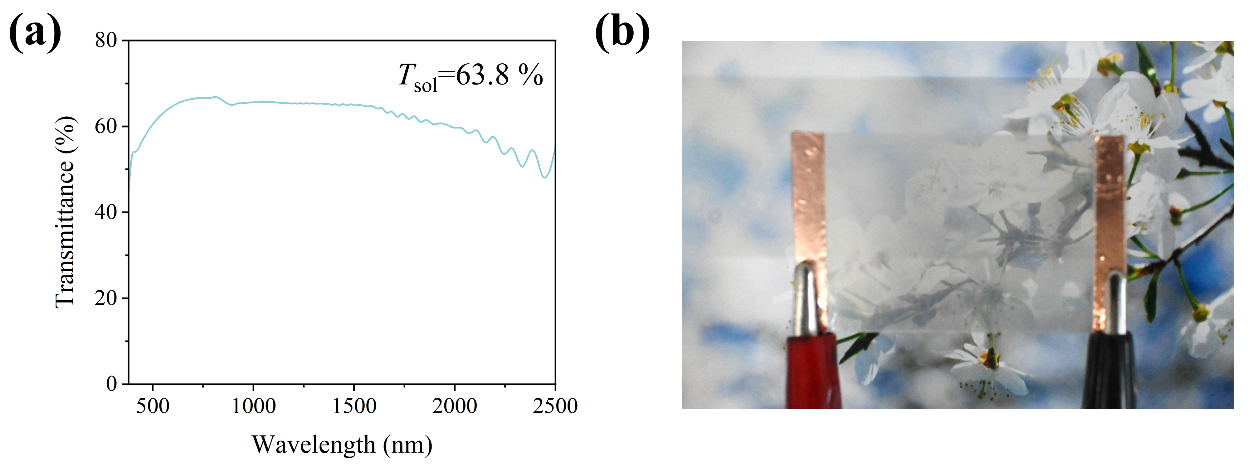


**Fig. S1** **a** Transmittance spectra of (0.38 – 2.5 µm) and **b** Photograph of the optimized BTMU at high solar transmittance (*T*_sol_) state


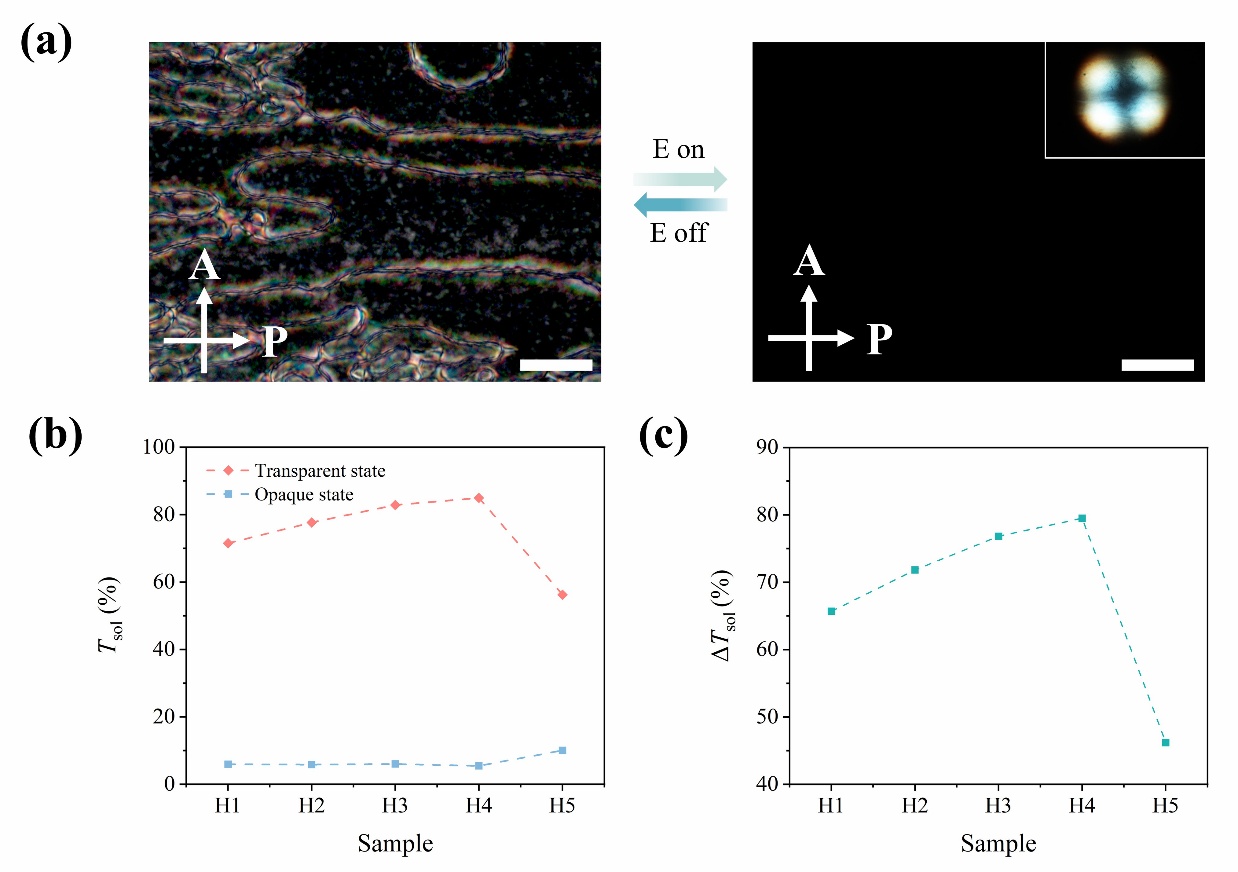


**Fig. S2** **a** Polarized optical microscope images of the small molecule liquid crystal without and under electrical field. The inset shows the cross-point. The scale bar is 40 μm. **b** Solar transmittance (*T*_sol_) the PDLCs with different contents of the A-HG at transparent (0 V) and opaque states (30 V). **c** Solar modulating ability (Δ*T*_sol_) the PDLCs with different contents of the A-HG


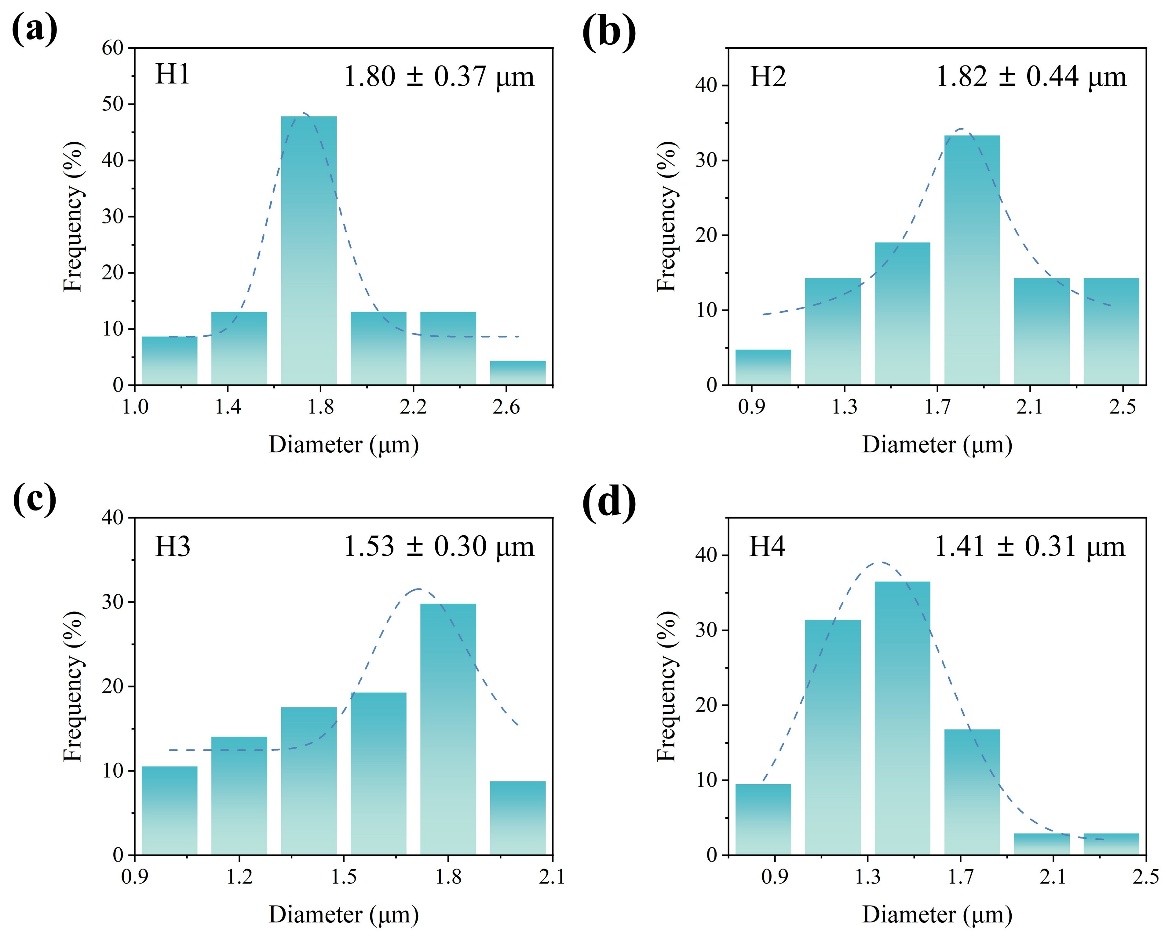


**Fig. S3** Size distribution of small molecule liquid crystal droplets in the PDLCs with different contents of the A-HG


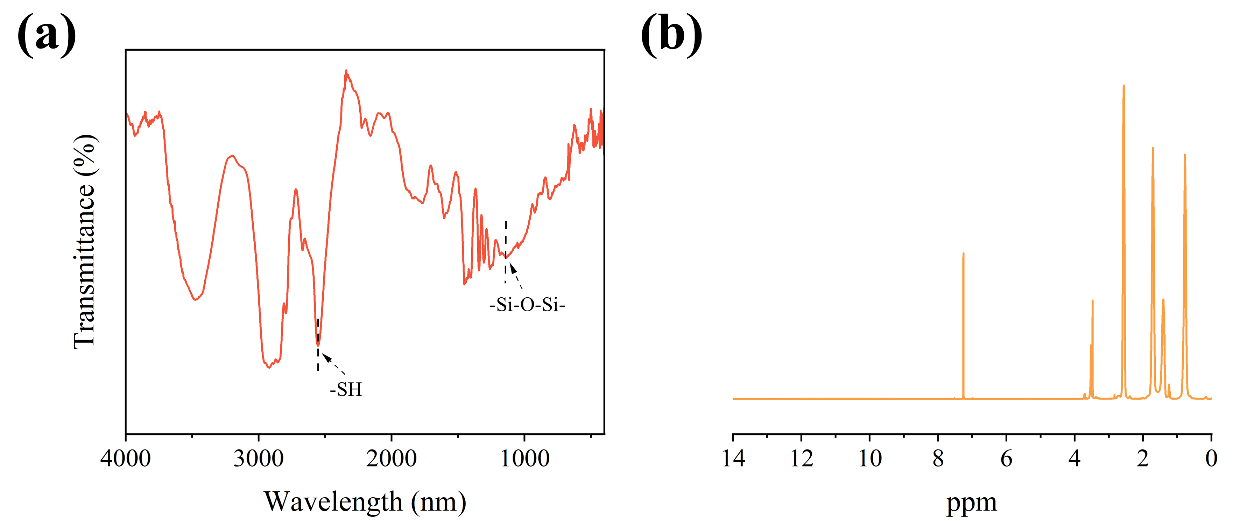


**Fig. S4** **a** Fourier transform tnfrared spectrometer (FTIR) spectra and **b** Nuclear magnetic resonance (NMR) spectroscopy of the POSS

**
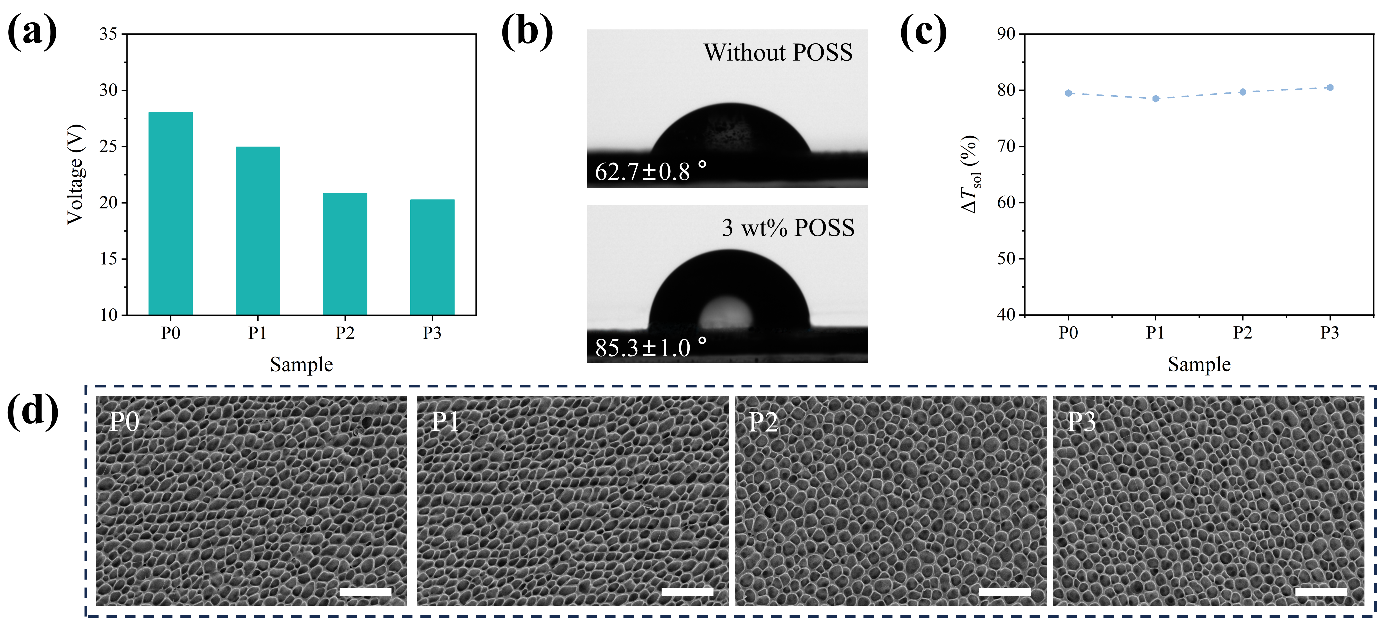
**

**Fig. S5** **a** Saturation voltage (V*_sat_*, driven voltage) of the PDLCs with different contents of the POSS. **b** Water contact angle of the polymer matrix with (3 wt%) and without non-polar POSS. **c** Solar modulating ability (Δ*T*_sol_), and **d** Scanning electron microscope (SEM) images of the PDLCs with different contents of the POSS. The scale bar is 10 μm

**
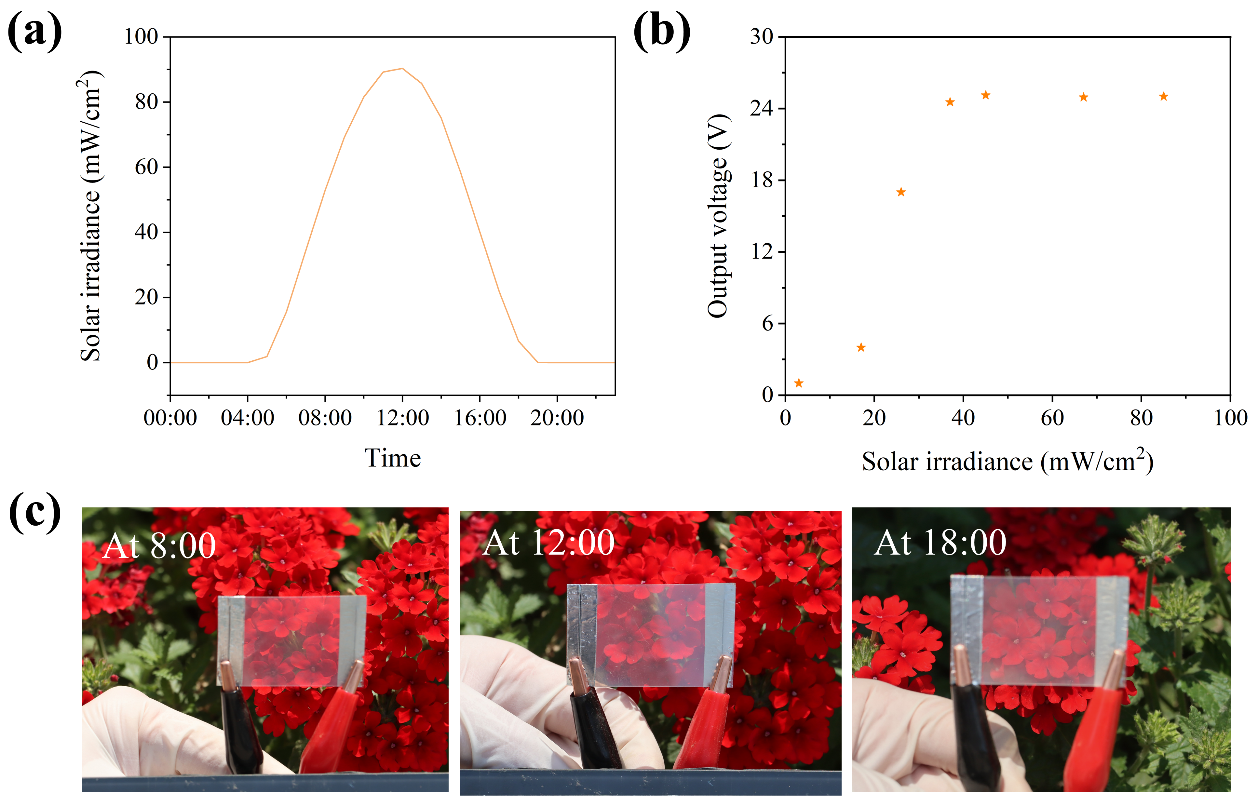
**

**Fig. S6** **a** Intensity of sunlight in a whole day at Beijing. **b** The output voltage curve of the perovskite solar cell under the irradiation of sunlight. **c** Photographs of the PDLCs under sunlight irradiation during daytime without applying a voltage


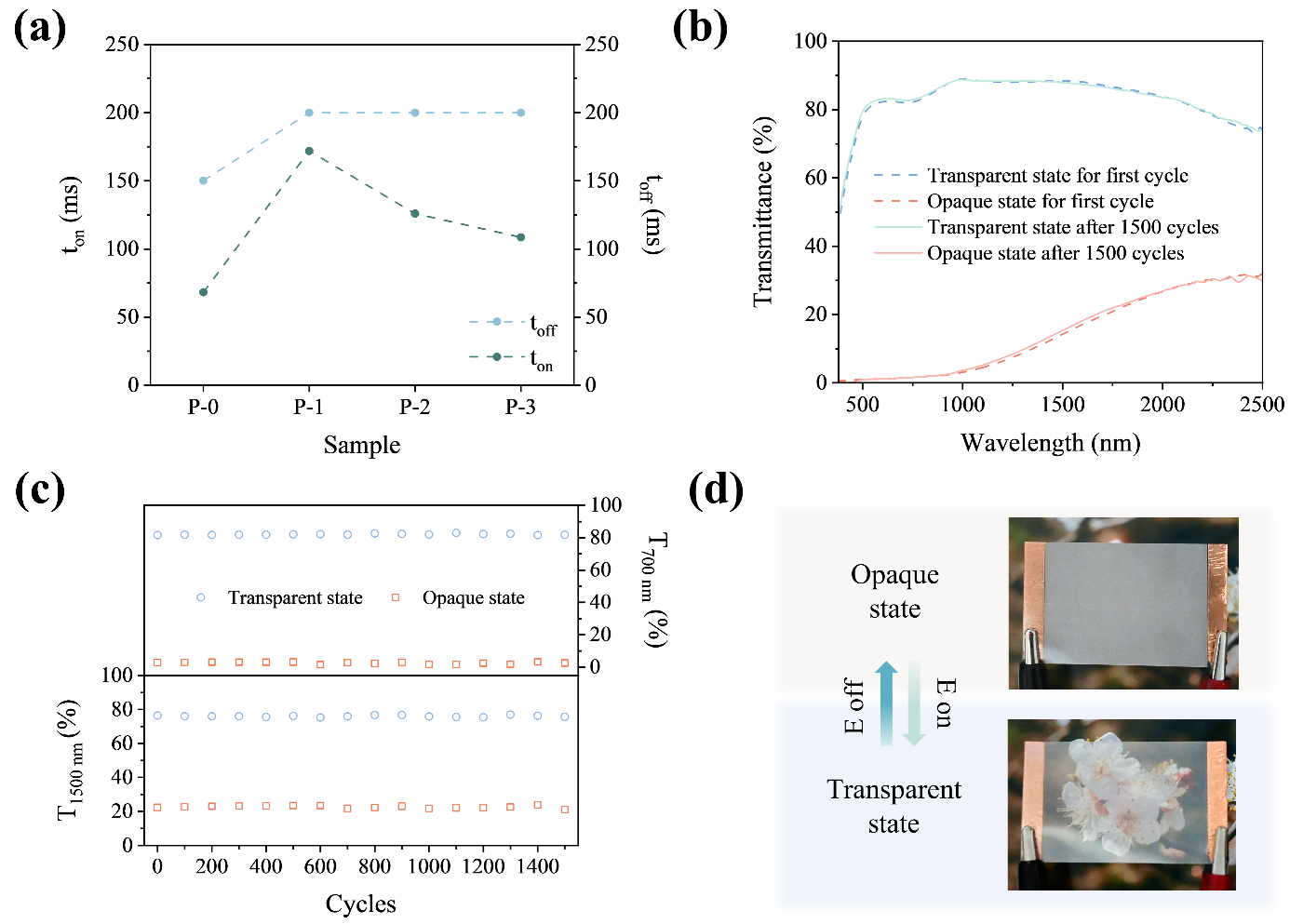


**Fig. S7** **a** Response time of the PDLCs. **b** UV-vis transmittance spectra of (0.38-2.5 µm) of the PDLCs (P3) at transparent (0 V) and opaque states (30 V) after 1500 cycles. **c** Transmittance variation curve of the PDLCs (P3) during 1500 cycles of the switching. **d** Photographs of the PDLCs (P3) at transparent (0 V) and opaque states (30 V) after 1500 cycles


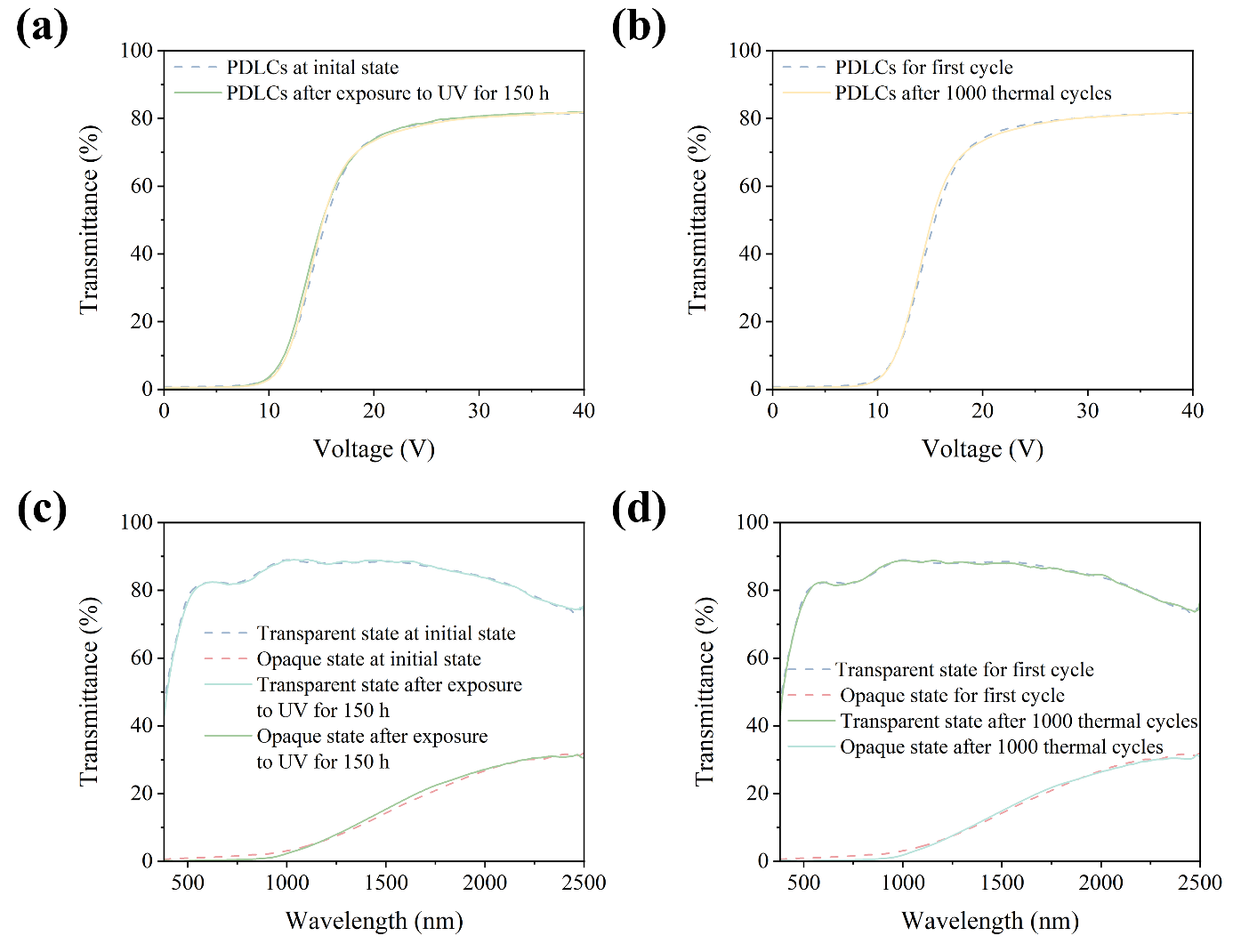


**Fig. S8** Environmental stability of the PDLCs (P3). Electro-optical curve of the PDLCs (P3) after **a** exposure to UV (150 W/m^2^) for 150 h and **b** 1000 times of thermal cycling (65/4 °C, 5 min hold at each temperature). UV-vis transmittance spectra of (0.38-2.5 µm) of the PDLCs (P3) at transparent (0 V) and opaque states (30 V) after **c** exposure to UV (150 W/m^2^) for 150 h and **d** 1000 times of thermal cycling (65/4 °C, 5 min hold at each temperature)


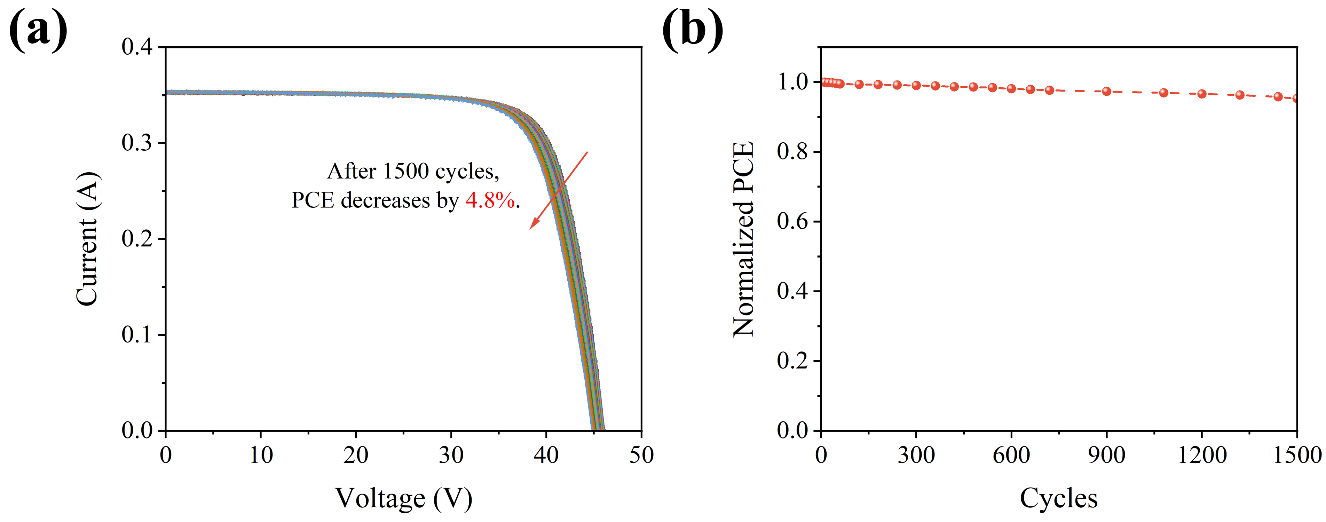


**Fig. S9** **a** Current-voltage (I-V) curves of the perovskite solar cell under PDLC switching stress. **b** The stability of the perovskite solar cell under PDLC switching stress


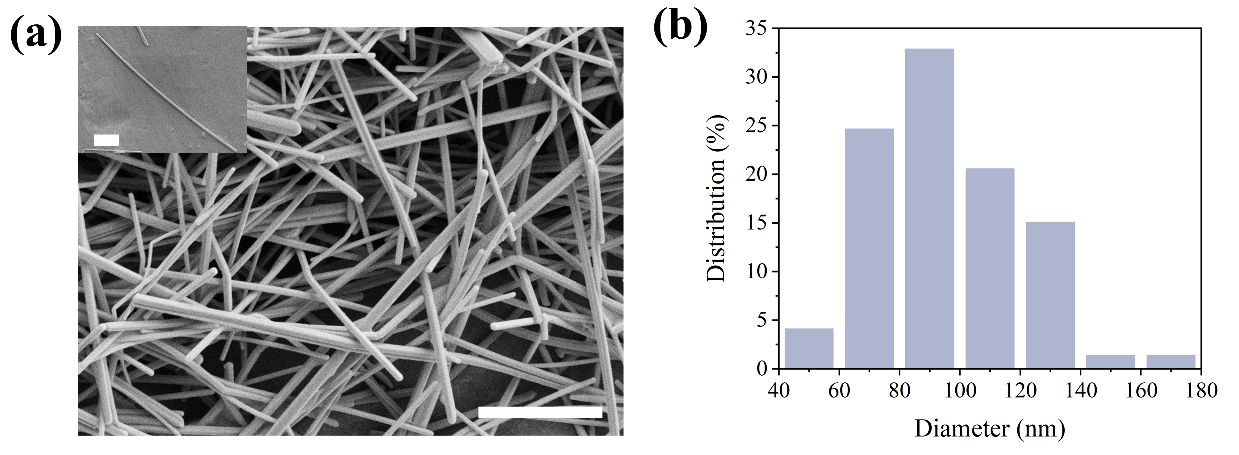


**Fig. S10** **a** SEM images of the lab synthesized Ag nanowires. Inset shows a single Ag nanowire. The scale bar is 2 μm. **b** Diameter distribution histogram of the Ag nanowires


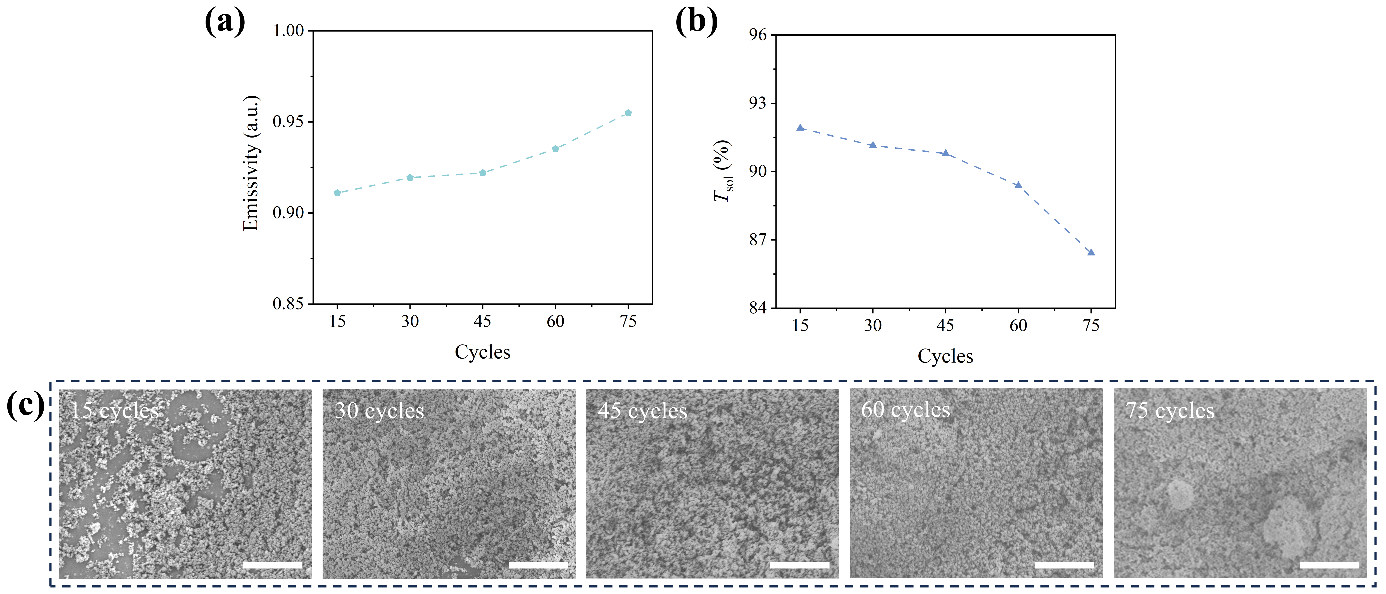


**Fig. S11** **a** Emissivity (*E*_MIR_) and **b** solar transmittance (*T*_sol_) the SiO_2_ PRC layer of different spraying cycle numbers. **c** SEM images of the surface of the SiO_2_ PRC layer of different spraying cycle numbers. The scale bar is 2 μm


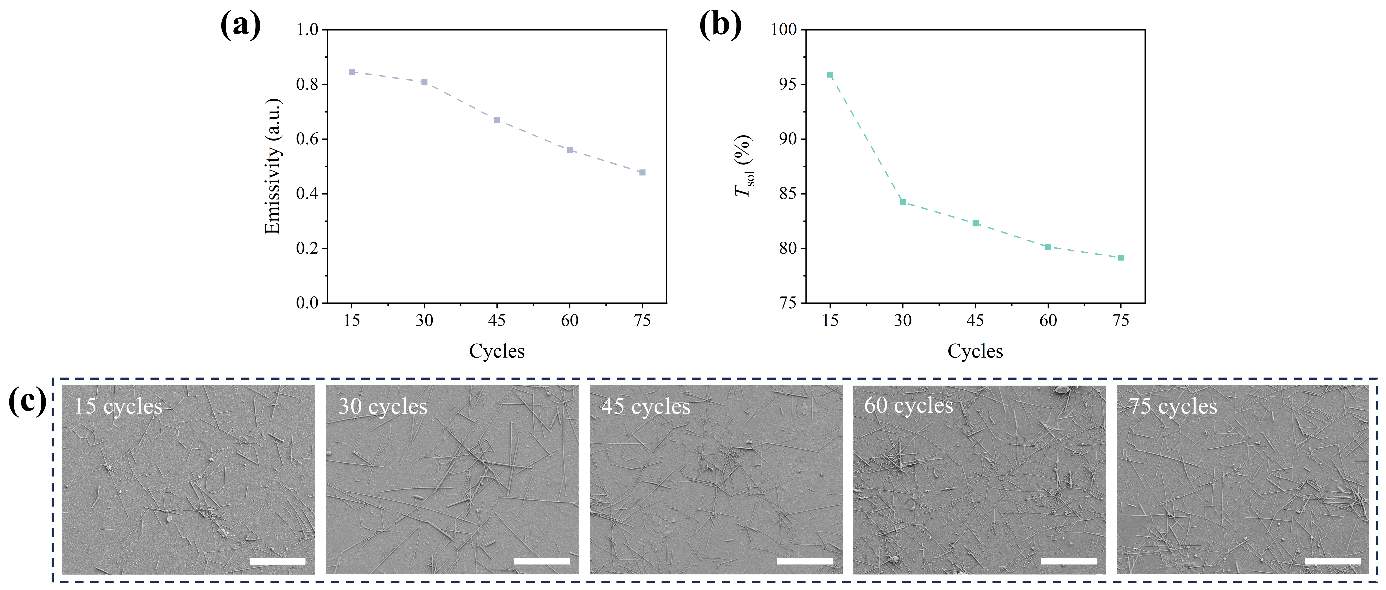


**Fig. S12** **a** Emissivity (*E*_MIR_) and **b** solar transmittance (*T*_sol_) the Ag layer of different spraying cycle numbers. **c** SEM images of the surface of the Ag layer of different spraying cycle numbers. The scale bar is 20 μm


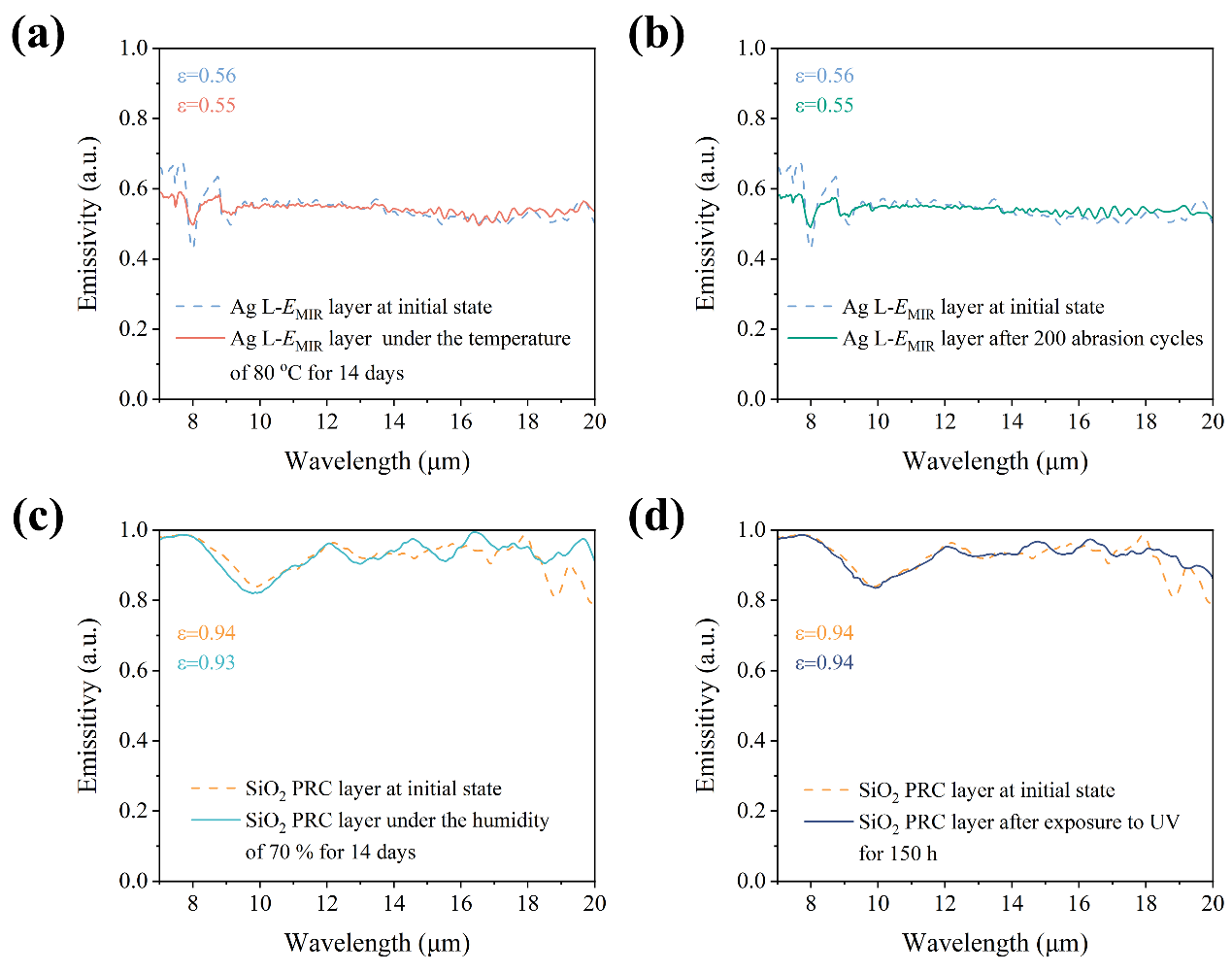


**Fig. S13** MIR emissivity (7.0-20 µm) of the Ag L-*E*_MIR_ layer (60 spraying cycles) after **a** put at high temperature 80 ^o^C for 14 days and **b** 200 abrasion cycles. MIR emissivity (7.0-20 µm) of the SiO_2_ passive radiation-cooling layer (60 spraying cycles) after **c** put under the humidity of 70 % (70 ^o^C) for 14 days and **d** exposure to UV (150 W/m^2^) for 150 h


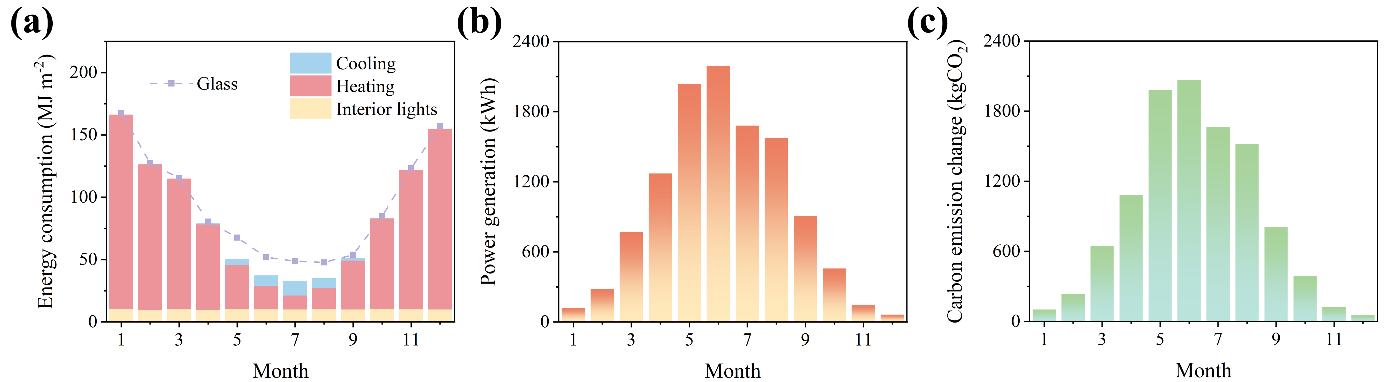


**Fig. S14 a** Monthly energy consumption of the SPWs and normal glass window in the climate condition of Oslo. **b** Monthly power generation of the SPWs according the working mode of the SPWs. **c** Monthly saving carbon dioxide emission estimated based on saving energy consumption and power generation of the SPWs


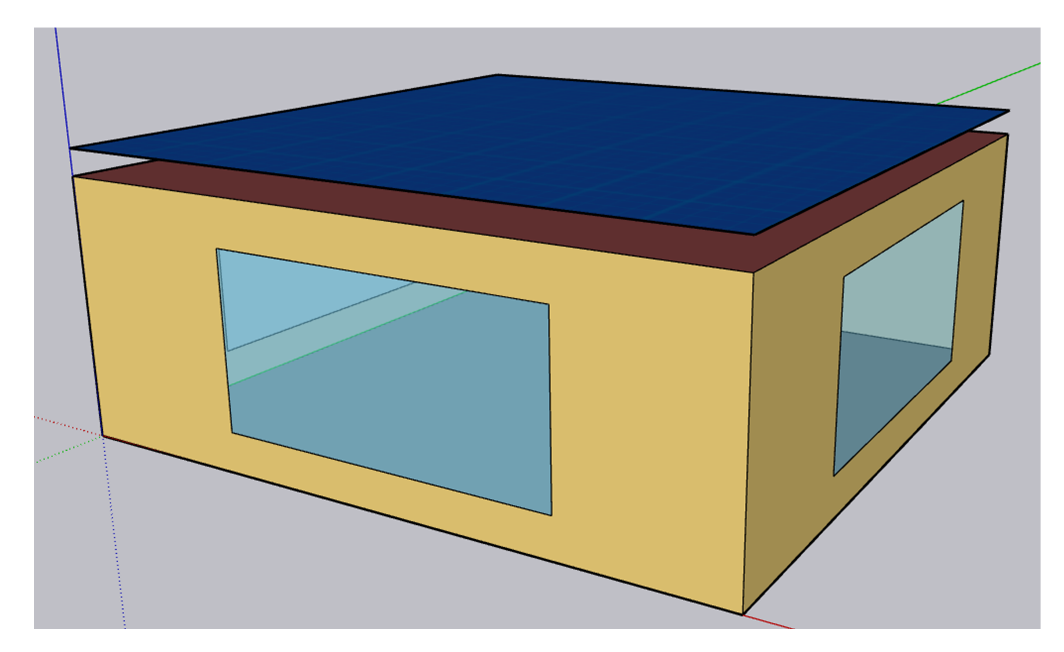


**Fig. S15** The house model for simulation. The house is 8 m (L) × 8 m (W) × 3 m (H). All four windows measure 4 m (L) × 2 m (W)

**Table S1** Detailed photovoltaic parameters of perovskites solar cell

| P_max_^a)^/ W | I_sc_^b)^/ A | V_oc_^c)^ / V | FF^d)^ / % | PCE^e)^ / % |
| --- | --- | --- | --- | --- |
| 12.22 | 0.40 | 44.61 | 68.77 | 16.29 |

^a)^P_max_: maximum power; ^b)^I_sc_: short-circuit current; ^c)^V_oc_: open-circuit voltage; ^d)^FF: fill factor; ^e)^PCE: power conversion efficiency.

**Table S2** Solar modulation performance comparison among state-of-the-art electrochromic smart window technologies

| References | Materials | Δ*T*_sol_ (400 nm-800 nm) / % |
| --- | --- | --- |
| **This work** | **PDLCs** | **76.6** |
| [S1] | WO_3_ | 72.9 |
| [S2] | WO_3_ | 31.4 |
| [S3] | WO_3_ | 48.4 |
| [S4] | WO_3-x_ | 60.3 |
| [S5] | WO_3_ | 79.9 |
| [S6] | WO_3_ | 11.5 |
| [S7] | WO_3_·H_2_O | 19.4 |
| [S8] | V_2_O_5_ | 28.1 |
| [S9] | PEDOT:PSS +  PANI-PAAMPSA | 46.8 |
| [S10] | PEDOT-MeOH | 17.2 |
| [S11] | HV(TF-SI)_2_ | 31.6 |
| [S12] | DPV | 43.0 |
| [S13] | PDLCs | 46.7 |
| [S14] | PDLCs | 45.5 |
| [S15] | PDLCs | 59.6 |
| [S16] | PDLCs | 58.3 |

**Table S3** The composition of PDLCs^a)^

| Sample | CHMA / wt% | HPMA / wt% | BDDA / wt% | PEGDA600 / wt% |
| --- | --- | --- | --- | --- |
| H1 | 27.25 | 0.00 | 2.07 | 8.18 |
| H2 | 24.90 | 3.39 | 1.79 | 7.51 |
| H3 | 22.46 | 6.67 | 1.69 | 7.76 |
| H4 | 19.97 | 10.05 | 1.50 | 6.01 |
| H5 | 17.26 | 13.43 | 1.32 | 5.26 |

^a)^In all samples, the weight ratio of E8, Irgacure 651 and glass microballoon (20 μm) is fixed at 60.00 wt%, 2.00 wt% and 0.50 wt%, respectively.

**Table S4** The composition of PDLCs with POSS-SH^a)^

| Sample | CHMA /wt% | HPMA /wt% | BDDA /wt% | PEGDA600 /wt% | POSS-SH  /wt% |
| --- | --- | --- | --- | --- | --- |
| P0 | 19.97 | 10.05 | 1.50 | 6.01 | 0.00 |
| P1 | 19.44 | 9.78 | 1.46 | 5.85 | 1.00 |
| P2 | 18.90 | 9.51 | 1.42 | 5.69 | 2.00 |
| P3 | 18.37 | 9.25 | 1.38 | 5.53 | 3.00 |

^a)^In all samples, the weight ratio of E8, Irgacure 651 and glass microballoon (20 μm) is fixed at 60.00 wt%, 2.00 wt% and 0.50 wt%, respectively.

**Supplementary Movies**

**Movie S1** Electrical-responsive behavior of the PDLCs (H4). The movie is played at 1X speed

**Movie S2** Alteration of the alignment of the small molecule liquid crystal (SMLC) under electrical field. The movie is played at 1X speed

**Movie S3** Self-powered behavior of the PDLCs (P3). The movie is played at 1X speed

**Movie S4** Cycling stability of the PDLCs (P3). The movie is played at 1X speed

**Supplementary References**

1. M.K. Ganesha, I. Mondal, A.K. Singh, G.U. Kulkarni, Fabrication of large-area, affordable dual-function electrochromic smart windows by using a hybrid electrode coated with an oxygen-deficient tungsten oxide ultrathin porous film. ACS Appl. Mater. Interfaces **15**(15), 19111–19120 (2023). <https://doi.org/10.1021/acsami.2c22638>
2. I. Mondal, M.K. Ganesha, A.K. Singh, G.U. Kulkarni, Affordable smart windows with dual-functionality: electrochromic color switching and charge storage. Adv. Mater. Technol. **8**(18), 2300651 (2023). <https://doi.org/10.1002/admt.202300651>
3. L. Lavagna, G. Syrrokostas, L. Fagiolari, J. Amici, C. Francia et al., Platinum-free photoelectrochromic devices working with copper-based electrolytes for ultrastable smart windows. J. Mater. Chem. A **9**(35), 19687–19691 (2021). <https://doi.org/10.1039/D1TA03544D>
4. M.K. Ganesha, H. Hakkeem, I. Mondal, A.K. Singh, G.U. Kulkarni, An ITO free all tungsten-based electrochromic energy storage device as smart window. Small **20**(48), 2405467 (2024). <https://doi.org/10.1002/smll.202405467>
5. M.K. Ganesha, H. Hakkeem, A.K. Singh, Redox potential based self-powered electrochromic devices for smart windows. Small **20**(42), e2403156 (2024). <https://doi.org/10.1002/smll.202403156>
6. R. Roy, R. Greeshma, A. Basith, R. Banerjee, A.K. Singh, Self-rechargeable aqueous Zn^2+/^K^+^ electrochromic energy storage device *via* scalable spray-coating integrated with Marangoni flow. Energy Storage Mater. **71**, 103680 (2024). <https://doi.org/10.1016/j.ensm.2024.103680>
7. R. Roy, R. Greeshma, P. Dutta, I. Mondal, R. Banerjee et al., Electrochromic and energy storage performance enhancement by introducing jahn–teller distortion: experimental and theoretical study. ACS Appl. Mater. Interfaces **16**(30), 39539–39550 (2024). <https://doi.org/10.1021/acsami.4c04445>
8. C. Wang, X. Zhang, S. Liu, H. Zhang, Q. Wang et al., Interfacial charge transfer and zinc ion intercalation and deintercalation dynamics in flexible multicolor electrochromic energy storage devices. ACS Appl. Energy Mater. **5**(1), 88–97 (2022). <https://doi.org/10.1021/acsaem.1c02508>
9. N.C. Davy, M. Sezen-Edmonds, J. Gao, X. Lin, A. Liu et al., Pairing of near-ultraviolet solar cells with electrochromic windows for smart management of the solar spectrum. Nat. Energy **2**, 17104 (2017). <https://doi.org/10.1038/nenergy.2017.104>
10. S. Yang, J. Zheng, M. Li, C. Xu, A novel photoelectrochromic device based on poly(3, 4-(2, 2-dimethylpropylenedioxy)thiophene) thin film and dye-sensitized solar cell. Sol. Energy Mater. Sol. Cells **97**, 186–190 (2012). <https://doi.org/10.1016/j.solmat.2011.09.038>
11. Y. Liu, J. Wang, F. Wang, Z. Cheng, Y. Fang et al., Full-frame and high-contrast smart windows from halide-exchanged perovskites. Nat. Commun. **12**(1), 3360 (2021). <https://doi.org/10.1038/s41467-021-23701-z>
12. H. Ling, J. Wu, F. Su, Y. Tian, Y.J. Liu, Automatic light-adjusting electrochromic device powered by perovskite solar cell. Nat. Commun. **12**(1), 1010 (2021). <https://doi.org/10.1038/s41467-021-21086-7>
13. Y. Deng, Y. Yang, Y. Xiao, H.-L. Xie, R. Lan et al., Ultrafast switchable passive radiative cooling smart windows with synergistic optical modulation. Adv. Funct. Mater. **33**(35), 2301319 (2023). <https://doi.org/10.1002/adfm.202301319>
14. Y. Deng, Y. Yang, Y. Xiao, X. Zeng, H.-L. Xie et al., Annual energy-saving smart windows with actively controllable passive radiative cooling and multimode heating regulation. Adv. Mater. **36**(27), 2401869 (2024). <https://doi.org/10.1002/adma.202401869>
15. Z. Zhang, Y. Yang, C. Ma, M. Yu, J. Xu et al., Enhanced electro-optical and heat regulation of intelligent dimming films using the photovoltaic effect of p–n heterostructures. Adv. Funct. Mater. **34**(45), 2406858 (2024). <https://doi.org/10.1002/adfm.202406858>
16. L. Zhang, C. Zou, Y. Gao, M. Yu, H. Yang, A multistage modulated smart window based on fluorinated polymer/liquid crystal composites with passive radiative cooling characteristics. Compos. Part A Appl. Sci. Manuf. **195**, 108980 (2025). <https://doi.org/10.1016/j.compositesa.2025.108980>
